# 20 years of stemness: From stem cells to hypertranscription and back

**DOI:** 10.1016/j.stemcr.2025.102406

**Published:** 2025-02-06

**Authors:** Yun-Kyo Kim, Miguel Ramalho-Santos

**Affiliations:** 1 Program in Developmental and Stem Cell Biology, Hospital for Sick Children, Toronto ON M5G 0A4, Canada; 2 Lunenfeld-Tanenbaum Research Institute, Mount Sinai Hospital, Toronto ON M5T 3L9, Canada; 3 Department of Molecular Genetics, University of Toronto, Toronto ON M5G 1X5, Canada

**Keywords:** stemness, embryonic stem cells, adult stem cells, hypertranscription, regeneration, RNA-seq, cell number normalization, single-cell RNA-seq, absolute scaling

## Abstract

Transcriptional profiling of stem cells came of age at the beginning of the century with the use of microarrays to analyze cell populations in bulk. Since then, stem cell transcriptomics has become increasingly sophisticated, notably with the recent widespread use of single-cell RNA sequencing. Here, we provide a perspective on how an early signature of genes upregulated in embryonic and adult stem cells, identified using microarrays over 20 years ago, serendipitously led to the recent discovery that stem/progenitor cells across organs are in a state of hypertranscription, a global elevation of the transcriptome. Looking back, we find that the 2002 stemness signature is a robust marker of stem cell hypertranscription, even though it was developed well before it was known what hypertranscription meant or how to detect it. We anticipate that studies of stem cell hypertranscription will be rich in novel insights in physiological and disease contexts for years to come.

## Teaser

Kim and Ramalho-Santos provide a historical perspective starting from the first studies in stem cell transcriptomics in the early 2000s to our present day understanding of hypertranscription in stem cell biology. The authors revisit a stemness signature from 2002, describe how it foreshadowed stem cell hypertranscription, and discuss future directions in the field.

## Introduction

A central tenet of Biology is that all cells in the body have essentially the same genome but differ in the genes that are active versus silenced. The transcriptional profile of a particular cell type, i.e., the set of genes it expresses and their expression levels, provides a picture of the pathways active in that cell type and is often used to define it. Identifying the transcriptional profiles of different cell types, the aspects that are shared and those that are unique, is therefore of high interest. This is particularly the case in stem cell biology, where multiple cell types and states with varying degrees of differentiation co-exist in a dynamic manner and are related by lineage. The advent of genome sequencing paved the way for transcriptional profiling of stem cells and their progeny, first with microarrays, later with RNA sequencing (RNA-seq) of bulk cell populations, and more recently with single-cell RNA-seq (scRNA-seq). Here, we provide a perspective on the continuum from studies in stem cell transcriptomics in the early 2000s to our present day understanding of hypertranscription in stem cell biology. We revisit a stemness signature from 2002 (Ramalho-Santos et al., 2002) that served as a foundation for studies of stem cell transcriptomics in our lab and others. We re-analyze this signature in light of the latest insights on stem cell hypertranscription, discuss its implications and utility, and propose future directions in this field. For in-depth reviews of hypertranscription, we refer the readers to Bowry et al. (2021), Kim et al. (2024), and Percharde et al. (2017a).

### Circa 2002: The search for stemness gene signatures

The turn of the century was a time of very active search for the transcriptional profiles of stem cells. These studies were primarily carried out in the mouse, where embryonic stem cells and adult stem cells from different organs (bone marrow, intestine, skin, etc.) had been studied for decades. A few markers of different stem cells had been identified, but their transcriptional profiles remained unknown and difficult to probe. This began to change with the development of custom and commercial microarrays (DeRisi et al., 1997; Lockhart et al., 1996; Schena et al., 1995). Microarrays allowed for the first time the simultaneous interrogation of thousands of genes, even before the draft human and mouse genomes were published in 2001 (Lander et al., 2001) and 2002 (Chinwalla et al., 2002), respectively. Several groups used microarrays to compare stem cells with their differentiated progeny, and to compare different types of stem cells (Fortunel et al., 2003; Geschwind et al., 2001; Ivanova et al., 2002; Kelly and Rizzino, 2000; Phillips et al., 2000; Ramalho-Santos et al., 2002; Terskikh et al., 2001), among others. The results from these studies helped dispel some confusion in the field at the time, when it was speculated that all stem cells might in fact be very similar in transcriptional profiles and differentiation potential (Blau et al., 2001; Clarke et al., 2000; Jiang et al., 2002). On the contrary, it became clear that each type of stem cell expresses a distinct transcriptional profile with unique markers (Fortunel et al., 2003; Ivanova et al., 2002; Ramalho-Santos et al., 2002). Interestingly, some sets of genes were identified as upregulated in common among different stem cell populations and were called “stemness” genes or a stem cell “signature” (Ivanova et al., 2002; Ramalho-Santos et al., 2002). The level of overlap between sets of stemness genes identified by different groups was modest, possibly reflecting differences in methods for isolation of stem cells, differentiated cell populations used as controls, protocols to amplify and label mRNAs, type and quality of microarrays, and criteria for calling differential gene expression (Burns and Zon, 2002; Evsikov and Solter, 2003; Fortunel et al., 2003; Ivanova et al., 2002, 2003; Ramalho-Santos et al., 2002). Nevertheless, these studies provided important resources for stem cell biologists in subsequent years.

**Figure 1:**
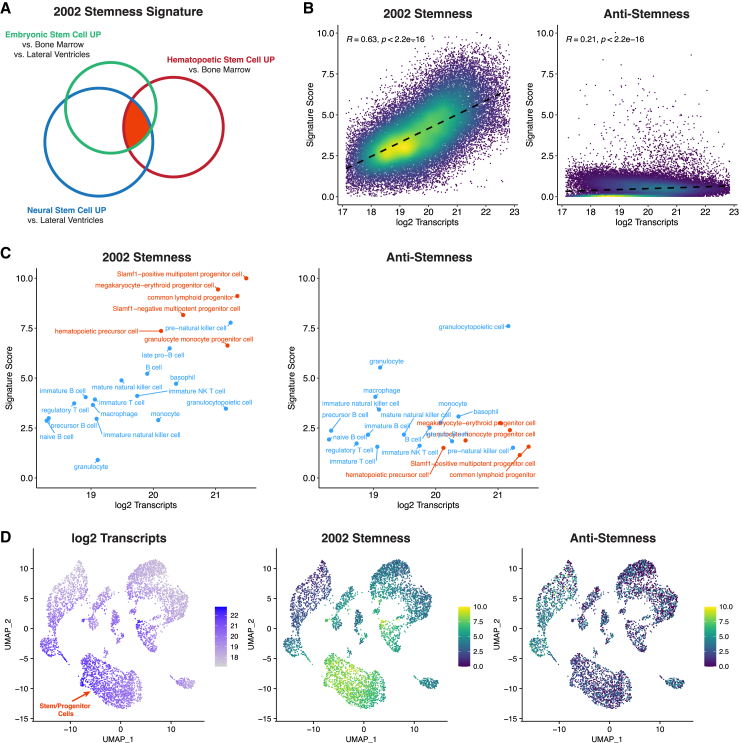
The stemness signature marks hypertranscribing hematopoietic stem and progenitor cells (A) Schematic of the original derivation of the 2002 stemness signature: overlap in genes upregulated in embryonic stem cells and hematopoietic and neural/stem progenitor cells (indicated in orange). Adapted from Ramalho-Santos et al. (2002). See Table S1 for the list of genes in this signature. (B) The stemness signature is strongly correlated with transcript content per cell in scRNA-seq data across mouse organs. Data from the Tabula Muris Consortium et al. (2018). A control gene set (“anti-stemness”) reported in the original study (Ramalho-Santos et al., 2002) is plotted, defined as genes upregulated in common in the differentiated tissues relative to the three stem cells. Signature enrichment scores were derived from globally scaled data, then plotted onto absolutely scaled transcript content data, as previously described (Kim et al., 2023). Spearman's correlation coefficients and *p* values are shown. (C) The stemness signature, but not the anti-stemness set, marks the various activated stem/progenitor cells in bone marrow data from Tabula Muris Consortium et al. (2018). (D) Uniform manifold approximation and projection (UMAP) of bone marrow data showing that hypertranscribing cells, which correspond to the stem/progenitor cells (Kim et al., 2023), are enriched for the stemness signature. A detailed annotation of cell types in this bone marrow dataset is provided in Tabula Muris Consortium et al. (2018).

A study led by one of us (M.R.-S.) determined the transcriptional profiles of mouse embryonic stem cells and adult hematopoietic and neural stem/progenitor cells and proposed a set of stemness genes upregulated in all three stem cell types (Ramalho-Santos et al., 2002) (Figure 1A, orange overlap). This signature (referred to as “2002 stemness signature” in this article) comprised genes involved in processes such as chromatin remodeling, DNA repair, translation, stress response and signaling, as well as several genes of unknown function at the time. None of these genes were detected as exclusively expressed in stem cells, but rather it was proposed that “their combined enrichment relative to differentiated cells (…) underlies the common properties of stem cells.” This proposal was highly speculative and difficult to test, as it was unknown which stemness genes were functional regulators of stem cells vs. “passenger” genes or false positives. Nevertheless, this work provided a resource that facilitated the subsequent identification of new stem cell populations and functional regulators. For example, the stemness signature contributed to the identification of mouse skin stem cells and human mammary stem/progenitor cells (Blanpain et al., 2004; Dontu et al., 2003). The stemness transcription factor Zfx was shown to be a regulator of embryonic stem cells, hematopoietic stem cells, and glioblastoma stem cells (Fang et al., 2014; Galan-Caridad et al., 2007). The transcription factor Yap and other members of the Hippo pathway were found to be key regulators of proliferation of embryonic, intestinal, skeletal, and neural stem cells (Camargo et al., 2007; Cao et al., 2008; Gregorieff et al., 2015; Judson et al., 2012; Lian et al., 2010; Qin et al., 2012; Tang et al., 2013). The chromatin remodeler chromodomain helicase DNA binding 1 (Chd1) (see the following section) was shown to regulate open chromatin and the transcriptional output of embryonic stem cells and nascent hematopoietic stem/progenitor cells (Gaspar-Maia et al., 2009; Guzman-Ayala et al., 2015; Koh et al., 2015). Regulatory roles for the deubiquitinase Usp9x were described in embryonic, hematopoietic, neural, and intestinal stem/progenitor cells (Jolly et al., 2009; Khan et al., 2018; Macrae and Ramalho-Santos, 2021; Naik et al., 2014; Premarathne et al., 2017). The metabolic regulator Gfer was shown to regulate embryonic and hematopoietic stem/progenitor cells (Teng et al., 2011; Todd et al., 2010). Resistance to oxidative stress was identified as a property shared by hematopoietic, neural, and mammary stem cells, as well as breast cancer stem cells (Diehn et al., 2009; Ito et al., 2004; Tsatmali et al., 2005). Several stemness genes were also shown to function in cellular reprogramming or regeneration (Gaspar-Maia et al., 2009; Hu et al., 2013; Lian et al., 2010; McClure et al., 2008). Despite these insights, it is important to note that many new regulators of stem cells were identified independently of the stemness signatures proposed by several groups. A good example is Lgr5, a G-coupled receptor that marks several epithelial stem cells in adult organs (Barker et al., 2007, 2010, 2012; Jaks et al., 2008; Ng et al., 2014; Plaks et al., 2013). Lgr5 was not part of the 2002 stemness signature because it is not expressed in embryonic stem cells (Ramalho-Santos et al., 2002).

### From stemness to open chromatin

Immediately after the identification of stemness signatures, the critical next step was to go beyond the accumulation of long lists of genes expressed in stem cells and identify those that might be key functional regulators. Genetic studies in mouse cells up to the turn of the century had been difficult and time-consuming, mostly assessing the function of one gene at a time by knockout approaches. The discovery and development of RNA interference (Fire et al., 1998) radically changed this. Several groups carried out RNA interference screens to identify novel stem cell regulators (Chia et al., 2010; Ding et al., 2009; Fazzio et al., 2008; Gaspar-Maia et al., 2009; Hu et al., 2009; Ivanova et al., 2006), mostly in embryonic stem cells because of their ease of manipulation in culture. In one such screen, we tested the function in mouse embryonic stem cells of ∼40 genes selected from various stem cell transcriptional profiling studies. These datasets included the stemness study (Ramalho-Santos et al., 2002) as well as analyses of the inner cell mass of the blastocyst, primordial germ cells, embryonic germ cells, and embryonic fibroblasts (Grskovic et al., 2007; Qin et al., 2012). This screen led to the identification of Chd1, which was part of 2002 stemness signature, as a regulator of the open chromatin state and self-renewal of embryonic stem cells (Gaspar-Maia et al., 2009). Work in the 2000s revealed that embryonic stem cells have a more open, decondensed chromatin (Ahmed et al., 2010; Meshorer et al., 2006; Park et al., 2004) and a globally elevated transcriptional output (Efroni et al., 2008) than differentiated cells, raising the question of how this state might be regulated.

Chd1 binds specifically to the activating histone mark H3K4me3 via its chromodomains (Flanagan et al., 2005) and acts to facilitate transcription (Sims et al., 2007). Chd1 appears to be the only member of the Chd family in which the chromodomains bind strongly to H3K4me3 (Flanagan et al., 2007). Chd1 also interacts directly with RNA polymerase II (RNA Pol) II and promotes removal of nucleosomal barriers to transcription (Farnung et al., 2021; Skene et al., 2014). In agreement with this notion, we found that Chd1 binding to chromatin in embryonic stem cells is highly correlated with H3K4me3, RNA Pol II, the transcription factor Myc, and gene expression (Gaspar-Maia et al., 2009). However, embryonic stem cells with Chd1 knocked down (Chd1 RNAi), or mutant for Chd1 (Chd1^−/−^) displayed essentially no downregulated genes in standard microarray or RNA-seq analyses (Gaspar-Maia et al., 2009; Guzman-Ayala et al., 2015). Moreover, a propensity of Chd1 RNAi embryonic stem cells to upregulate some neural genes (Gaspar-Maia et al., 2009) could be suppressed with improvements in cell culture conditions (Guzman-Ayala et al., 2015). Thus, embryonic stem cells lacking Chd1, a chromatin factor strongly associated with transcriptional activation, appeared to express a largely unchanged transcriptome, even though they displayed reduced self-renewal and increased heterochromatin (Gaspar-Maia et al., 2009; Guzman-Ayala et al., 2015).

### Circa 2012: Cell-number normalization in transcriptomics

To state that a transcriptome is “unchanged,” of course, requires that we are able to reliably detect change. Most studies of transcriptomics up until 2012 assumed that all cells express approximately the same total amount of RNA per cell, with the majority of the genes remaining unchanged in expression. This assumption had arisen from a need to control for sources of artifacts in transcriptional profiling and had been very useful to identify cell type-specific transcripts. However, it effectively meant that, if one cell type expresses a globally higher or lower level of most of its mRNAs than another cell type, this difference would be masked (reviewed by Lovén et al. (2012)). Such standard normalization procedures, used to the present time, include hybridizing equal amounts of labeled RNA in microarrays (DeRisi et al., 1997; Lockhart et al., 1996; Schena et al., 1995) or read-depth normalization in RNA-seq (Mortazavi et al., 2008; Sultan et al., 2008; Wilhelm et al., 2008). Two studies in 2012, showing that overexpression of Myc leads to a global increase in transcriptional output in cancer cells and immune cells (Lin et al., 2012; Nie et al., 2012), prompted the exploration of alternative normalization methods. These kinds of global transcriptional shifts can be preserved if equal numbers of cells are processed and “spiked” with equal amounts of exogeneous synthetic RNAs, which are later bioinformatically used to scale the data to represent cell numbers (reviewed by Lovén et al. (2012) and Percharde et al. (2017a)). Similar “cell-number normalization” (CNN) approaches can be applied to many techniques, from western blotting and quantitative reverse-transcription PCR to RNA-seq and chromatin immunoprecipitation sequencing (ChIP-seq), among others (Percharde et al., 2017a). Thus, these methodologies provide opportunities to uncover hypertranscription, defined as a global elevation of the majority of the transcriptome at the nascent level, relative to neighboring cells, or earlier or later cell stages (Percharde et al., 2017a). This definition highlights the relative, context-dependent nature of hypertranscription (Kim et al., 2024; Percharde et al., 2017a).

### Developmental hypertranscription

Taking advantage of these new methodologies, we carried out CNN RNA-seq in Chd1^−/−^ embryonic stem cells (Guzman-Ayala et al., 2015). Remarkably, what before had been an “unchanged” transcriptome in the mutant cells proved instead to be a global reduction across thousands of mRNAs and non-coding RNAs (Guzman-Ayala et al., 2015). This downshift in transcriptional output in the mutant cells manifests also at the level of total RNA per cell and levels of ribosomal RNA (Bulut-Karslioglu et al., 2021; Guzman-Ayala et al., 2015). Moreover, the results align well with the arrest of Chd1^−/−^ mouse embryos immediately after implantation (E5.5–6.5) (Guzman-Ayala et al., 2015), a stage of very rapid growth (Snow, 1977). Already at E5.5, Chd1^−/−^ embryos display a drastic reduction in nascent output of rRNA and fewer epiblast cells than controls. Thus, both cell culture and *in vivo* data indicate that Chd1 is required for global transcriptional output at peri-implantation (Bulut-Karslioglu et al., 2021; Guzman-Ayala et al., 2015).

In subsequent years, global shifts of transcriptional output were explored in other contexts of development. For example, hypertranscription was found to occur during emergence of definitive hematopoietic stem/progenitor cells from hemogenic endothelium at mid-gestation (Koh et al., 2015). Cre-mediated deletion of Chd1 in the endothelium is compatible with normal vasculogenesis but leads to suppressed global transcriptional output in hemogenic beds and a loss of hematopoietic stem/progenitor cells (Koh et al., 2015). Here again, Chd1 is not required for transcription per se, but for a global elevation of transcriptional output at a critical developmental transition. Around the same time in development, the embryonic germline undergoes a remarkable level of hypertranscription, driven by N-Myc (Percharde et al., 2017b). There is evidence for hypertranscription persisting or recurring in the adult stages of gametogenesis, including the classical case of lampbrush chromosomes during oocyte growth (Callan, 1987; Simeoni et al., 2012; Xia et al., 2020). Moreover, hypertranscription occurs in neural progenitors during brain development, in this case mediated by the Yap/Taz transcription factors (Lavado et al., 2018). Conversely, we found that global downregulation of transcriptional output, or hypotranscription, is a feature of developmental pausing (Bulut-Karslioglu et al., 2016). Clearly, much remains to be explored on developmental hypertranscription regarding where it occurs, how it is regulated, and what functions it serves in each context.

### Circa 2022: Hypertranscription in adult stem/progenitor cells

The instances of hypertranscription during development raised the question of whether it might also occur in adulthood. The high heterogeneity of cell types in adult organs represents an obstacle to analyses of transcriptional profiles in bulk assays. This obstacle has been removed with the advent of scRNA-seq methodologies, which in turn has spurred large-scale efforts to compile multi-organ single-cell “atlases” (Tabula Muris Consortium et al., 2018). Traditional scRNA-seq analysis employs global scaling normalization which, similar to read-depth normalization in bulk RNA-seq, masks hypertranscription. To overcome this, we developed absolute scaling approaches to unmask global shifts in transcriptional output in scRNA-seq data, taking advantage of features that are commonly implemented in single-cell library preparation (Kim et al., 2023). These features, including unique molecular identifiers and exogenous spike-ins, can be used to estimate differences in transcript content between thousands of single-cell transcriptomes, effectively being the equivalent for scRNA-seq of what CNN is for bulk RNA-seq (Kim et al., 2023).

Using absolute scaling, we recently explored the extent of hypertranscription across adult organs, during homeostasis and regeneration (Kim et al., 2023). This analysis revealed a wide diversity of cellular transcript content at both inter- and intra-organ levels. We found that elevated transcriptional output is a remarkably consistent feature of adult multipotent stem and progenitor cells across organs, most notably within tissues characterized by rapid cellular turnover (bone marrow, intestine, and skin) (Kim et al., 2023). This capacity for hypertranscription is associated with multilineage potential: acquisition of terminal identities during differentiation is accompanied by a decline in transcriptional output. In the case of muscle (Oprescu et al., 2020), quiescent stem cells (muscle satellite cells) have low transcriptional output, but their activation in response to injury and regeneration triggers rapid upregulation of global transcription (Kim et al., 2023). Interestingly, the increase in transcriptional output in muscle stem cells occurs 1–2 days before they increase in number, suggesting that hypertranscription may fuel the biosynthetic demand of muscle repair. Hypertranscribing stem/progenitor cells in adult organs are enriched for pathways previously found associated with developmental hypertranscription, such as chromatin remodeling, DNA repair, translation, RNA processing, and the mTor and Myc pathways (Kim et al., 2023; Percharde et al., 2017a). Similarly to embryonic stem cells, a more open, decondensed chromatin state may also be a feature of activated adult stem/progenitor cells (Boonsanay et al., 2016; Parmentier et al., 2022; Reddien and Sánchez Alvarado, 2004; Spangrude et al., 1988). These results indicate that hypertranscription is a shared feature of embryonic and adult stem/progenitor cells during development, organ homeostasis, and regeneration (Kim et al., 2024).

### The 2002 stemness signature foreshadowed stem cell hypertranscription during homeostasis and regeneration

The recurrence of hypertranscription in stem/progenitor cells of diverse embryonic and adult origins led us to question whether stemness, defined in a broad transcriptomic sense, may be associated with transcriptional output in stem cell compartments. The 2002 stemness signature, which set the stage for later findings on stem cell hypertranscription (as discussed above), comprises ∼200 genes upregulated in embryonic stem cells and adult hematopoietic and neural stem/progenitor cells (Ramalho-Santos et al., 2002). The most current annotation of gene symbols in this signature is provided in Table S1. Notably, this study was carried out with standard normalization of microarray data, therefore precluding analyses of global transcriptional shifts. Here, we revisited the 2002 stemness signature (Figure 1A) by calculating globally scaled signature scores to be superimposed on scRNA-seq datasets, including the multi-organ Tabula Muris cell atlas (Tabula Muris Consortium et al., 2018). As a control, we used a gene set, also reported in the 2002 study, of genes with the opposite expression pattern: upregulated in common in differentiated cells (whole bone marrow and lateral ventricular brain tissue), relative to the three stem cell populations analyzed. This control gene set is denoted here as “anti-stemness.” By calculating the signature scores based on globally scaled rather than absolutely scaled data (i.e., normalizing gene expression in a relative manner assuming all cells express about the same amount of RNA per cell), the score values are independent of any underlying global transcriptional shifts. In other words, the score values represent a relative enrichment of a gene set in each cell that is “blind” to hyper/hypotranscription, much like Gene Ontology or gene set enrichment analysis (Subramanian et al., 2005). Remarkably, we found a strong correlation of the expression of the 2002 stemness signature, but not anti-stemness, with global transcript levels across the entire cell atlas (Figure 1B). When applied specifically to the bone marrow data within the Tabula Muris study, the 2002 stemness signature correctly identifies the hematopoietic stem/progenitor cells and is strongly associated with the absolute transcriptional output of blood cell types (Figures 1C and 1D).

**Figure 2:**
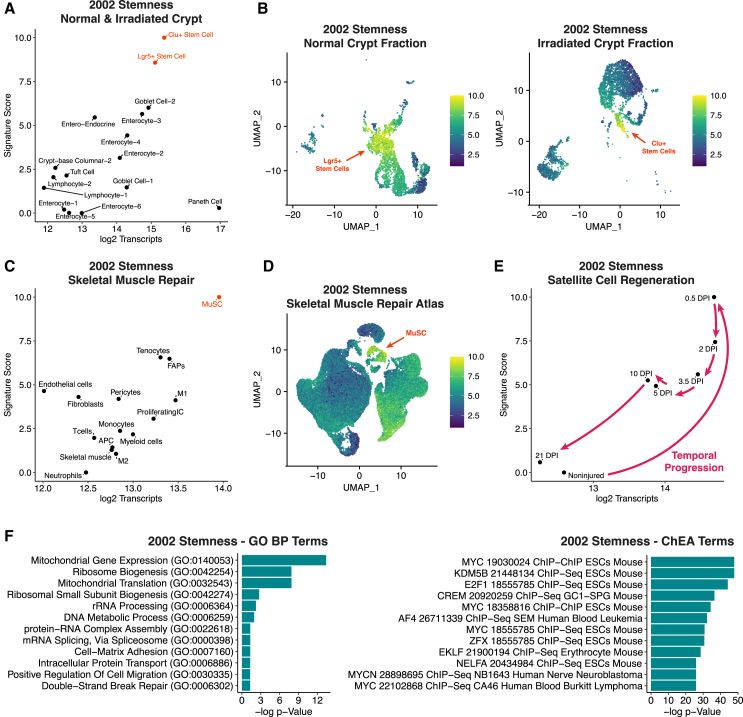
The stemness signature is enriched in intestinal and muscle stem cells during regeneration (A) Stem cells that drive physiological (Lgr5+) or injury-induced (Clu+) renewal of the intestinal epithelium are strongly enriched for the stemness signature. Note the low enrichment of Paneth cells for the stemness signature, despite their very high transcript content. Data from Ayyaz et al. (2019). (B) UMAP plots highlighting the enrichment for the stemness signature in Lgr5+ intestinal stem cells and Clu+ revival stem cells. (C) Enrichment for the stemness signature in muscle stem cells. Data from Oprescu et al. (2020). The muscle stem cell data (MuSC) include both quiescent and regenerating cells (see below). (D) UMAP plots displaying the enrichment for the stemness signature in muscle stem cells. Detailed annotations of cell types in the intestinal and muscle datasets are provided in Kim et al., 2023. (E) The stemness signature is activated in muscle stem cells during injury-induced regeneration, correlating with their transcript content. DPI: days post injury. Neither intestinal nor muscle stem cells were part of the samples used to derive the stemness signature (Figure 1A and [Ramalho-Santos et al., 2002]). (F) The stemness signature is enriched for genes with roles in mitochondrial gene expression, ribosome biogenesis, splicing, cell adhesion, and DNA repair (GO BP, Gene Ontology Biological Process), and for chromatin targets of Myc transcription factors and other proteins associated with hypertranscription (ChEA, ChIP enrichment analysis). GO analyses and ChEA were carried out as before (Kim et al., 2023).

We next tested the predictive power of the stemness signature when applied to scRNA-seq datasets from contexts of regeneration (Figure 2). In intestinal crypts, Lgr5+ intestinal stem cells support physiological renewal of the epithelium and have high transcriptional output (Kim et al., 2023). Upon gamma-irradiation, regeneration is driven by alternative Clu+ “revival” stem cells (Ayyaz et al., 2019), which are also in state of hypertranscription (Kim et al., 2023). Strikingly, Lgr5+ intestinal stem cells and Clu+ revival stem cells are the cell types with the strongest enrichment for the stemness signature in the intestine (Figures 2A and 2B). It is important to note that the stemness signature is not simply reflecting the high transcriptional output of these cells: Paneth cells, a terminally differentiated cell type, have the highest absolute transcript levels per cell in the intestine, but negative enrichment for the stemness signature (Figure 2A). In other words, the stemness signature does not generally capture all hypertranscription, but rather stem cell hypertranscription.

Similar observations can be made in skeletal muscle (Oprescu et al., 2020): as mentioned earlier, stem cells are activated in response to injury, with concurrent elevation of transcriptional output (Kim et al., 2023). Muscle stem cell activation is marked by a strong upregulation of the stemness signature (Figures 2C–2E). With ongoing muscle regeneration, gradual loss of transcriptional output in the muscle stem cells is paralleled closely by a gradual decrease in expression of the stemness signature (Figure 2E). Note that quiescent muscle stem cells prior to injury or after regeneration is complete are not enriched for the stemness signature (Figure 2E). Thus, the stemness signature primarily captures the activated stem cell state, one of high biosynthetic demand, likely because this state constituted the major fraction of cells used in the study where this signature was defined (Ramalho-Santos et al., 2002). These findings highlight the robustness of the stemness signature to identify activated stem/progenitor cells in organs not included in the original 2002 study (Ramalho-Santos et al., 2002), such as the intestine and muscle (Figures 2A–2E).

It is clear that the stemness signature is a composite of several cellular functions, not simply hypertranscription. As mentioned earlier, there are hypertranscribing cells, such as intestinal Paneth cells, that are not stem cells and are not enriched for the stemness signature (Figure 2A). Moreover, while the stemness signature is often associated with proliferating cells, it does not simply reflect cell proliferation. For example, several classes of lymphocytes in the bone marrow score highly for cell cycle markers (Kim et al., 2023) but not for the stemness signature (Figure 1C). The correlation between hypertranscription and cell cycle is itself modest (Kim et al., 2023). Thus, the stemness signature reflects several pathways enriched in activated stem/progenitor cells, notably hypertranscription and cell cycle, but likely also others, such as DNA repair, proteostasis, metabolism, or stress responses. Each of these pathways is not by any means exclusive to activated stem/progenitor cells; it is rather their combined enrichment that may define these cells, as already noted in the 2002 study that put forth the stemness signature (Ramalho-Santos et al., 2002).

It is important to note that the pervasive occurrence of hypertranscription across stem cell compartments, or the property of hypertranscription for that matter, was not evident from the composition of the 2002 stemness signature at the time. As described earlier, CNN approaches were not developed until 2012. Moreover, ChIP-seq studies to identify chromatin targets of factors later associated with hypertranscription, like Myc, had yet to be carried out. In hindsight, it is now clear that some molecular features of the stemness signature are reflective of hallmark hypertranscription pathways as currently defined. Gene Ontology analysis of the stemness gene set reveals an enrichment of functional terms such as mitochondrial gene expression, ribosome biogenesis, splicing, cell adhesion, and DNA repair (Figure 2F), consistent with the upregulation of these pathways in hypertranscribing cells (Kim et al., 2023; Percharde et al., 2017a, 2017b, 2017a). The association of hypertranscription with translation is not simply due to a co-occurrence of both processes in stages of biosynthetic demand and growth, but rather a functional co-dependence. Hypertranscription in embryonic stem cells is itself acutely dependent on translational output, because the euchromatin regulators that drive hypertranscription, including Myc factors and Chd1, are preferentially unstable proteins (Bulut-Karslioglu et al., 2018). Moreover, chromatin enrichment analysis shows that the stemness signature is overrepresented for targets of Myc and other factors with roles in global transcriptional activation (Figure 2F). Interestingly, while no Myc family members are part of the stemness signature, different Myc genes were detected as upregulated in each of the stem/progenitor cell populations originally profiled (N-Myc in embryonic and neural stem/progenitor cells, and c-Myc in hematopoietic stem/progenitor cells) (Ramalho-Santos et al., 2002). Thus, the stemness signature contained within it hallmarks of stem cell hypertranscription, but it took 20 years of progress in functional studies, CNN methodologies, and absolute scaling in scRNA-seq data to appreciate that.

## Conclusions and future directions

In this piece, we provided a historical perspective of how early transcriptomics approaches to define stemness contributed to findings on developmental hypertranscription, which in turn paved the way for the discovery that hypertranscription is pervasively redeployed in adult stem cell compartments (Kim et al., 2023). The insights described here document that the 2002 stemness signature can be used as a marker, though by no means the only one, to help identify activated stem/progenitor cells across organs. In particular, this signature may provide a transcriptional readout of the level of activation of stem/progenitor cells at different time points during ontogeny or upon genetic or environmental perturbation. Of note, this signature captures the activated stem/progenitor cell state even when no information is available on absolute transcriptional output, e.g., in standard read-depth normalized bulk RNA-seq data or globally scaled scRNA-seq data (Figures 1B, 2B, and 2D). As described earlier (Figure 2E), the stemness signature is not an adequate tool to identify rare, quiescent stem cells. It will be of interest to explore how this signature can be modified or augmented with the incorporation of other stem cell gene expression datasets or analysis pipelines. For example, using methods for inferring or tracing cell lineage in scRNA-seq (La Manno et al., 2018; Wagner and Klein, 2020), it may be possible to identify candidate slower cycling, reserve stem cells: these are expected to score lowly for the stemness signature but give rise to cells that score highly for it. In addition, the stemness signature can be used in parallel with transcriptomic metrics of multilineage potential that have recently been described (Gulati et al., 2020; Zhang et al., 2020). Importantly, none of these potential signature correlations replace functional studies of stem cell activity.

We anticipate that the years ahead will prove fruitful for the dissection of the molecular regulation of stem cell hypertranscription and how it is integrated with aspects such as metabolic activity, proteostasis, DNA repair, cell cycle, cell size, and the balance between self-renewal and differentiation (for detailed discussions, see Kim et al. (2024) and Percharde et al. (2017a)). It will also be of interest to study the function of stem cell hypertranscription at the tissue and organ level. While in many cases hypertranscription may primarily support the high biosynthetic demand of organ growth and regeneration, it is likely that it has additional roles. For example, pervasive transcription may protect the genome of stem cells from accumulation of mutations, similar to what has been reported during spermatogenesis (Xia et al., 2020). Moreover, it is clear that hypertranscription includes the upregulation of vast non-coding portions of the genome, such as repeats and transposable elements, which have regulatory potential (Babarinde et al., 2021; Efroni et al., 2008; Fort et al., 2014; Martens et al., 2005). Hypertranscription may also play a functional role in cases of regeneration where differentiated cells revert to a stem cell-like state (Cho et al., 2024) or during induced cellular reprogramming (Babos et al., 2019; Zviran et al., 2019). Finally, studies of stem cell hypertranscription are anticipated to provide novel insights in disease contexts where hypertranscription has been recently reported, such as cancer (Bowry et al., 2021; Cao et al., 2022; Zatzman et al., 2022) and aging (Debès et al., 2023; Hu et al., 2014).

## Acknowledgments

We thank Janet Rossant, Gordon Keller, Jeff Wrana, Gary Bader, Aydan Bulut-Karslioglu, anonymous reviewers, and members of the Santos lab for critical reading of the manuscript. We are grateful to past and present members and collaborators of our lab. We thank S. Yoon, Y. Matsuzaki, R.C. Mulligan, W.H. Wong, and D.A. Melton for their contribution to the 2002 stemness signature study. We apologize to colleagues whose work may not have been cited due to space constraints. Y.-K.K. is supported by Whiteside (University of Toronto), Vanier (CIHR), and McLaughlin (University of Toronto) scholarships. Work in the Santos lab is supported by CIHR Project grants 165935 and 178094 and a Canada 150 Research Chair in Developmental Epigenetics.

## Author contributions

M.R.-S. and Y.-K.K. conceived the project. Y.-K.K. performed all data analysis, with supervision by M.R.-S. Y.-K.K. and M.R.-S. wrote the manuscript.

## Declaration of interests

The authors declare no competing interests.

## Supplemental information

Table S1. Updated annotation of gene symbols in the stemness 2002 signature, related to Figure 1 The Affymetrix U74Av2 microarrays used consisted of 3 sets of chips: chip A contained probes for the best annotated genes at the time, whereas chips B and C consisted mostly of probes for expressed sequence tags of unknown genes. In the original publication (Ramalho-Santos et al., 2002), the stemness signature was obtained using chip A. For consistency with the original work, this signature derived from chip A (“Stemness A”, 214 genes) was used throughout this piece. To facilitate future studies, we also provide the stemness signature from combined analysis of chips A, B and C (“Stemness ABC”, 314 genes), which yields very similar results to Stemness A in the analyses described in Figures 1 and 2.
